# Can nutritional and inflammatory indices predict 90-day mortality in fragility hip fracture patients?

**DOI:** 10.1051/sicotj/2023029

**Published:** 2023-10-30

**Authors:** Tal Frenkel Rutenberg, Avital Hershkovitz, Rana Jabareen, Maria Vitenberg, Efrat Daglan, Moti Iflah, Michael Drexler, Shai Shemesh

**Affiliations:** 1 Department of Orthopaedic Surgery, Rabin Medical Center 39 Jabotinsky st. Petach Tikva 4941492 Israel; 2 Sackler Faculty of Medicine, Tel Aviv University, Ramat Aviv Tel Aviv 6423906 Israel; 3 Beit Rivka Geriatric Rehabilitation Center 4 Ha Hamisha st. Petach Tikva 4924577 Israel and Sackler Faculty of Medicine, Tel Aviv University, Ramat Aviv Tel Aviv 6423906 Israel; 4 Department of Orthopaedic Surgery, Samson Assuta Ashdod University Hospital and Faculty of Health Sciences, Ben Gurion University 7, Ha’Refua Street 7747629 Ashdod Israel

**Keywords:** Fragility hip fracture, Survival, Nutrition, Inflammation, Platelet-to-lymphocyte ratio

## Abstract

*Introduction*: Hip fractures in the elderly are related to increased mortality. The identification of patients at risk is essential. Several nutritional and inflammatory parameters were investigated in an effort to find a prognostic indicator for mortality following fragility hip fractures (FHF) surgery. We aim to evaluate their utility and compare between the different factors. *Methods*: A retrospective cohort study of patients 65 years and older, who underwent surgery following fragility hip fractures between January 2012 and June 2020, was conducted. Patients who died within 90 days were matched at a 1:1 ratio with surviving controls, based on age, gender, fracture type, and comorbidities. Nutritional and inflammatory indices, including serum albumin, protein energy malnutrition (PEM), albumin-to-globulin ratio (AGR), prognostic nutritional index (PNI), the systemic immune-inflammation index (SII), platelet-to-lymphocyte ratio (PLR), and the neutrophile-to-lymphocyte ratio (NLR), were compared between groups. *Results*: 304 patients were included, 152 in each group. Patients’ demographics were similar. Among all indices evaluated, only the PLR significantly differed between the study groups (236.9 ± 193.5 for the study group vs. 186.6 ± 119.3 for the control group (*p* = 0.007). In patients who survived the initial hospitalization, the PEM was also found to be correlated with 90 days mortality. *Discussion*: The PLR was found to be correlated with mortality risk following FHF surgery. As it can be easily calculated from accessible blood tests, we recommend its’ routine assessment as a screening tool for personalized management of patients at high risk for mortality.

## Introduction

Hip fractures in the elderly are related to increased morbidity, reduced mobility and independence, high mortality rates, and high treatment costs [1, 2]. Malnutrition is prevalent in elderly patients who present with fragility hip fractures (FHF) [3]. It is associated with worse patient outcomes, increased complication rate, lower rates of regained previous mobility, and increased mortality [4–7].

Several nutritional and inflammatory parameters were investigated in an effort to find a prognostic indicator for mortality and complications following fragility hip fractures. The most extensively explored parameter is serum albumin. Albumin, produced by the liver, is the most substantial plasmatic protein [5]. It is affected by the nutritional status, and not by age itself [8]. Hypoalbuminemia was found by many to be related to elevated mortality rates following FHFs [1, 4, 9–14]. Other researchers [15–18] combined serum albumin with total lymphocyte count to calculate the Protein Energy Malnutrition (PEM) indicator and found it to be associated with reduced one-year survival following FHFs. Finally, the albumin-to-globulin ratio (AGR) was found to be related to reduced survival in cancer patients [19, 20].

Additional parameters considered as prognostic factors following fragility hip fracture surgery are the prognostic nutritional index (PNI), an index easily calculated using the following equation: [(10 × serum albumin (g/dL)) + (0.005 × total lymphocyte count)] [21], the Systemic immune-inflammation index (SII) based on peripheral platelet, neutrophil, and lymphocyte counts (platelet × total neutrophil count/total lymphocyte count) [10], the platelet-to-lymphocyte ratio (PLR) [22], and the neutrophile to lymphocyte ratio (NLR) [23, 24].

In this study, we aimed to evaluate and compare the utility of all the above prognostic indicators for the prediction of post-operative 90-mortality risk in FHF patients.

## Material and methods

### Study design

Following approval of the institution review board, a retrospective cohort study of patients 65 years and older, who underwent surgery following fragility hip fractures (AO classification of fractures: 31A1, 31A2, 31A3, 31B1, 31B2, and 31B3) [25, 26] between January 2012 and June 2020, in a single orthopedic department was conducted. Patients who died within 90 days of surgery were matched with a 1:1 ratio with surviving controls based on age, gender, Carlson co-morbidity index (CCI) [27], and fracture type (intracapsular and extracapsular). Surgical therapy was defined as closed reduction, open reduction, or hemiarthroplasty. Exclusion criteria were pathological and impending fractures, fractures sustained during a hospital stay due to other medical causes, and neglected hip fractures. For patients who presented with a contralateral fracture during the study period, only the first fracture was regarded as the index procedure.

The primary outcome measure was 90-day mortality. Secondary outcome measures were: length of stay (LOS) and in-hospital infections.

### Data collection

Demographic data, walking ability, and living arrangements were collected. The age-adjusted CCI (ACCI) [26, 27], was used to evaluate patients’ comorbidities. Regular anticoagulation treatment was noted. Hospitalization characteristics such as time to surgery (defined as the time from admission to the operating room), fracture type, length of hospital stay, in-hospital complications, and pre-surgical laboratory values (white blood count, total lymphocyte count, neutrophils, hemoglobin, platelets, creatinine, albumin, globulin, and international normalized ratio [INR]) were gathered. If a chemistry laboratory result was not available at admission, a value from the preceding month was gathered (albumin and globulin, as well as blood count, INR, and basic chemistry are routinely drawn at admission).

Data was collected via shared electronic medical records software, which allows access to hospitalization data from all hospitals in the community.

### Statistical analysis

Continuous variables are presented as mean and standard deviation (*SD*). Quantitative and ordinal variables are presented as absolute and relative frequencies. The Student’s *T*-test was used to compare numeric variables and the Fisher’s exact tests for categorical variables. All reported *p*-values are two-tailed. Statistical significance was defined as *p* < 0.05. Statistical analysis was performed using R [28].

## Results

One thousand five hundred forty-six patients aged 65 years and older were presented with proximal hip fracture during the study period and met the inclusion criteria; of whom, 152 died within 90 days of surgery and were matched with a similar number of controls.

Demographic features are presented in Table 1. Patient demographics and co-morbidities were similar between groups. Fifty-three patients (35% of the study group) have died during the index hospitalization, while the other 99 have died 47.2 ± 23 days following discharge.

**Table 1:** Patients’ baseline characteristics.

Variable	90-days mortality (*n* = 152)	Control (*n* = 152)	*P*-value
Age, average (*SD*)	86 (7.2)	86 (7.2)	0.995
Gender, *n* (%)			
Male	63 (41.4)	66 (43.4)	0.817
Charlson’s comorbidity index, average (*SD*)	3.4 (2.3)	3.3 (3.2)	0.556
Fracture type, *n* (%)			
Extracapsular	83 (45.6)	82 (53.9)	1
Intracapsular	69 (45.4)	70 (46.1)	
Walking aid, *n* (%)*			
None	69 (47.6)	57 (38.5)	0.151
Cane	24 (16.6)	32 (21.6)	
Walker	52 (35.9)	56 (37.8)	
Wheelchair	0	3 (2)	
Living arrangement, *n* (%)**			
Home	84 (56)	97 (63.8)	0.391
Nursing home	25 (16.7)	21 (13.8)	
Home with caregiver	41 (27.3)	34 (22.4)	
Hospitalization department, *n* (%)			
Orthopedics	60 (39.5)	71 (46.7)	0.467
Geriatrics	88 (57.9)	77 (50.6)	
Medicine	4 (2.6)	4 (2.6)	
Anticoagulation use, *n* (%)	33 (21.7)	27 (17.6)	0.472
Laterality, *n* (%)			
Right	67 (44.1)	74 (48.7)	0.490

* Data was unavailable for seven patients from the study group and for four patients from the control group.

** Data was unavailable for two patients from the study group.

Time to surgery was similar for the entire cohort (28.5 ± 33 h). The rate of in-hospital postoperative infections (urinary tract infection, pneumonia, sepsis, and pressure sores) differed between groups, and all were more prevalent in the study group (Table 2). LOS was similar in both groups, 10.4 ± 6.7 days.

**Table 2:** Perioperative clinical and laboratory data.

	90-days mortality (*n* = 152)	Control (*n* = 152)	*P*-value
Time to surgery (hours), average (*SD*)	31 (37.6)	26.1 (27.6)	0.203
Length of stay (days) average (*SD*)	10.9 (7.5)	9.9 (5.8)	0.151
Blood units given, average (*SD*)	0.9 (1.2)	1 (1)	0.622
Laboratory values at admission, average (*SD*)			
Hemoglobin, gr/dl	11.6 (1.8)	11.9 (1.5)	0.213
Platelet count, ×10^9^/L	235.3 (105)	220.8 (80.3)	0.177
Lymphocytes, ×10^9^/L	2.5 (4)	2.4 (3.8)	0.912
Neutrophils, ×10^9^/L	8 (4.2)	8.5 (4.5)	0.327
Creatinine, mg/dL	1.3 (1)	1.2 (0.7)	0.231
In-hospital postoperative infections, *n* (%)			
Urinary tract infection	13 (8.6)	3 (2)	0.018
Pneumonia	26 (17.1)	4 (2.6)	<0.001
Sepsis	13 (8.6)	3 (2)	0.018
Pressure sores	5 (3.5)	4 (2.6)	1

Nutritional indices results are presented in Table 3. Of all indices estimated, only one was found to significantly differ between the study groups, the platelets to lymphocytes ratio, which was significantly higher in the study group (236.9 ± 193.5 vs. 186.8 ± 119.3, *p* = 0.007). This was also true when excluding patients who were affected with a postoperative systemic infection (245.8 ± 214 for the study group vs. 182.2 ± 115.6 for the other group, *p* = 0.006) and when excluding patients who died during the index hospitalization (239.4 ± 193.8 for the study group vs. 186.6 ± 119.3 for the other group, *p* = 0.019). Notably, when the nutritional indices were calculated only for patients who have survived the initial hospitalization, the PEM index was also found to significantly differ between groups, as 18.6% of the study group patients and 7.5% of patients from the other group, met with the reduced albumin and lymphocyte counts thresholds (*p* = 0018).

**Table 3:** Nutritional indices.

Variable	90-days mortality (*n* = 152)	Control (*n* = 152)	*P*-value
Albumin, average (*SD*)*	3.7 (0.5)	3.8 (0.5)	0.220
Albumin to globulin ratio, average (*SD*)*	1.4 (0.4)	1.5 (0.3)	0.153
Prognostic nutritional index, average (*SD*) *	49.8 (21.6)	49.6 (18.9)	0.938
Protein energy malnutrition index, *n* (%)*	29 (21.2)	20 (15)	0.212
Systemic immune-inflammation index, average (*SD*)	2100.8 (2463.5)	1781.9 (1618.2)	0.183
Neutrophile to lymphocyte ratio, average (*SD*)	8.6 (7.3)	8.1 (6.3)	0.543
Platelets to lymphocyte ratio, average (*SD*)	236.9 (193.5)	186.8 (119.3)	0.007

* Data was unavailable for 15 patients from the study group and for 19 patients from the control group.

## Discussion

Fragility hip fractures are associated with increased morbidity and mortality rates of up to 20–30% in the post-operative year [29]. The relationship between inflammation and adverse outcomes following FHF surgery is gaining attention [23, 24, 30], as well as the association between malnutrition and elderly FHF patients’ survival [16, 18, 31]. While many indices aim to detect patients at risk for reduced survival, we found only the platelets to lymphocytes ratio to correlate with 90 days postoperative mortality.

PLR was found to be associated with increased all-cause mortality in the elderly [32]. However, when looking specifically into the FHF patients’ population, results are conflicting. While some [22] have found PLR to be associated with increased 6–12 months mortality [33], others [34, 35] have failed to demonstrate this effect. In our cohort, PLR was associated with increased 90 mortality. This was also true when controlling for postoperative infections or in-hospital mortality. Wang et al. [22] have defined high PLR (≥189) to be associated with increased one-year all-cause mortality. Interestingly, although in this study PLR was analyzed as a continuous variable, the study group’s PLR average was indeed higher than the suggested value, and vice versa for the control group.

PEM is associated with an increased morbidity and mortality. It is seen more often in patients with FHF than in age-matched controls [3] and was found to be correlated with adverse outcomes in this patient population [17, 18, 36]. In our cohort, it was associated with increased 90 mortality in patients who survived the initial hospitalization. Eneroth et al. [3] found that daily nutritional supplements for merely 10 days following surgery in FHF patients with PEM reduced complication rates and mortality at 120 days following surgery. As poor nutritional status, impaired dietary intake, and deficient muscle health are common in older hip fracture patients in geriatric rehabilitation wards [37], our findings may further support nutritional supplements for this frail patient population.

Finally, while PEM significantly differed between groups, the PNI, which is also based on albumin and lymphocyte counts, did not differ between groups. This discrepancy is probably related to the different ways in which the index is calculated. While the PEM is related to patients with hypoalbuminemia of <3.5 g/dL and lymphopenia of <1.5 cells/mm^3^ [16] the PNI is calculated by multiplying albumin and lymphocytes values (10 × ALB g/dL + 0.005 × lymphocyte count cells/mm^3^ [38]). This means that the PEM may identify patients who are more severely malnourished, and only this particular patient population was found to be at increased risk for mortality.

Other nutritional and inflammatory indices were not found to be correlated with increased 90 days mortality. The role of albumin, an important serum protein that reflects the patient’s metabolic status, was found by others [5, 12, 13, 15, 17], but not in our cohort, to be associated with post-operative mortality. Globulins are the other major component of total serum proteins that play a major role in immunity and inflammation [39]. While the AGR was found to predict survival and disease progression in the field of oncology [19, 20, 39, 40], we did not find such a correlation in the FHF patient population.

The SII has been considered to be a promising prognostic predictor in various diseases, including coronary artery disease, ischemic stroke, and in the field of oncology [10]. So far, only Wang et al. [10] have explored its relation with survival following FHF surgery and found it to be correlated with one-year mortality. While SII was higher in the study group, in our cohort no significant difference between groups was found. Finally, another inflammatory index, the NLR, which was found by some researchers to predict mortality following FHF surgery [23, 24, 34], was also not found to be indicative of mortality in our group. This was also true when comparing patients with NLR of 8.5 and higher, a value which was found to mostly correlate with post-operative morbidity and mortality by Fisher et al. [24].

Several limitations of this study should be considered. Firstly, as our study is observational, the inference of a causal relationship between admission inflammatory and nutritional indices and outcomes is limited. Secondly, although patients were matched according to age and CCI, it might be that specific comorbidity may lead to an impairment of the immune system more than others, leading to an unpredictable effect on the indices calculated. Finally, the rate of in-hospital mortality was high (35%) and in-hospital infectious complications which often result in patient demise were higher in the study group. However, when patients with in-hospital infections and mortality were excluded, the PLR still significantly differed between groups.

In conclusion, of the several nutritional and inflammatory indices hypothesized to predict mortality following fragility hip fractures, only the PLR indicator was found to correlate with 90 days mortality. Its calculation is straightforward, as it is based on blood exams which are routinely obtained on admission and may provide important information on patients who are at high risk for mortality. Multi-center prospective studies are required to explore whether interventions to lower PLR values can potentially improve outcomes.
